# RECOMIA—a cloud-based platform for artificial intelligence research in nuclear medicine and radiology

**DOI:** 10.1186/s40658-020-00316-9

**Published:** 2020-08-04

**Authors:** Elin Trägårdh, Pablo Borrelli, Reza Kaboteh, Tony Gillberg, Johannes Ulén, Olof Enqvist, Lars Edenbrandt

**Affiliations:** 1 Department of Clinical Physiology and Nuclear Medicine, Skåne University Hospital, Carl Bertil Laurells gata 9, 205 02 Malmö, Sweden; 2 Wallenberg Centre for Molecular Medicine, Lund University, Lund, Sweden; 3 Department of Clinical Physiology, Sahlgrenska University Hospital, Gothenburg, Sweden; 4 RECOMIA, Malmö, Sweden; 5 Eigenvision AB, Malmö, Sweden; 6 Department of Electrical Engineering, Chalmers University of Technology, Gothenburg, Sweden; 7 Department of Molecular and Clinical Medicine, Institute of Medicine, Sahlgrenska Academy, University of Gothenburg, Gothenburg, Sweden

**Keywords:** CNN, Artificial intelligence, Deep learning, Segmentation, PET-CT

## Abstract

**Background:**

Artificial intelligence (AI) is about to transform medical imaging. The Research Consortium for Medical Image Analysis (RECOMIA), a not-for-profit organisation, has developed an online platform to facilitate collaboration between medical researchers and AI researchers. The aim is to minimise the time and effort researchers need to spend on technical aspects, such as transfer, display, and annotation of images, as well as legal aspects, such as de-identification. The purpose of this article is to present the RECOMIA platform and its AI-based tools for organ segmentation in computed tomography (CT), which can be used for extraction of standardised uptake values from the corresponding positron emission tomography (PET) image.

**Results:**

The RECOMIA platform includes modules for (1) local de-identification of medical images, (2) secure transfer of images to the cloud-based platform, (3) display functions available using a standard web browser, (4) tools for manual annotation of organs or pathology in the images, (5) deep learning-based tools for organ segmentation or other customised analyses, (6) tools for quantification of segmented volumes, and (7) an export function for the quantitative results. The AI-based tool for organ segmentation in CT currently handles 100 organs (77 bones and 23 soft tissue organs). The segmentation is based on two convolutional neural networks (CNNs): one network to handle organs with multiple similar instances, such as vertebrae and ribs, and one network for all other organs. The CNNs have been trained using CT studies from 339 patients. Experienced radiologists annotated organs in the CT studies. The performance of the segmentation tool, measured as mean Dice index on a manually annotated test set, with 10 representative organs, was 0.93 for all foreground voxels, and the mean Dice index over the organs were 0.86 (0.82 for the soft tissue organs and 0.90 for the bones).

**Conclusion:**

The paper presents a platform that provides deep learning-based tools that can perform basic organ segmentations in CT, which can then be used to automatically obtain the different measurement in the corresponding PET image. The RECOMIA platform is available on request at www.recomia.org for research purposes.

## Background

Artificial intelligence (AI) is about to transform the field of medical imaging. Deep learning, a subfield of AI, has become the method of choice for image analysis applications. This technique provides new opportunities in developing tools for automated analysis of 3-dimensional computed tomography (CT), positron emission tomography (PET)/CT, and magnetic resonance imaging. These tools have the potential to improve or substitute current methods of assessing CT, PET/CT, and magnetic resonance imaging in patients with cancer, for example, the Response Evaluation Criteria in Solid Tumors and PET Response Evaluation Criteria in Solid Tumors [1–3]. The development of these approaches is, however, hindered by technical and legal aspects that the researchers need to spend time and effort on. A platform for communication, image transfer, and analysis could minimise these problems.

The Research Consortium for Medical Image Analysis (RECOMIA) is a not-for-profit organisation that aims to promote research in the fields of AI and medical imaging. RECOMIA has developed a cloud-based platform to facilitate collaboration between medical researchers focusing on patient images and the related information, and mathematical researchers developing deep learning-based tools. The aim is to minimise the time and effort researchers need to spend on technical aspects, such as transfer and display of digital imaging and communications in medicine (DICOM) images and image annotations, as well as legal aspects, such as de-identification, and compliance with the General Data Protection Regulation and the Health Insurance Portability and Accountability Act.

Deep learning-based tools can be trained to analyse medical images using images with manual annotations of organs or pathology, such as tumours. The RECOMIA platform provides deep learning-based tools that can perform organ segmentations in CT, detection of lesions in PET/CT, and automated quantitative analysis of the segmented/detected volumes. These tools are freely available for researchers on reasonable request at www.recomia.org. At present, more than 100 different organs and lesions can be segmented/detected based on training databases consisting of CT and PET/CT studies. This article aims to present the RECOMIA platform and the status of the current deep learning-based CT tools.

## Material and methods

### Platform

**Fig. 1:**
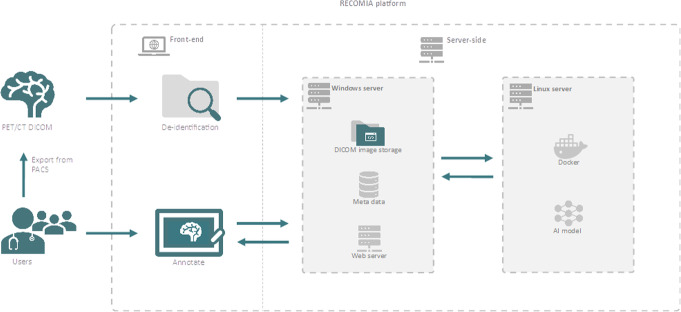
RECOMIA platform architecture overview and user interaction

The RECOMIA platform is a cloud-based platform running on two separate servers (Fig. 1). One is a Windows server running the web application handling everything but the AI models; it is written in C# using the ASP.NET Framework. One is a Linux server running Docker handling the AI models. To simplify collaboration between researchers at different universities and hospitals in different countries, the platform requires no installation and all functionality is available from a standard web browser. For security, the platform is deployed in an ISO/IEC 27001-certified data centre, and the recommended hardening, such as IP restrictions, is applied.

#### De-identification and upload

**Fig. 2:**
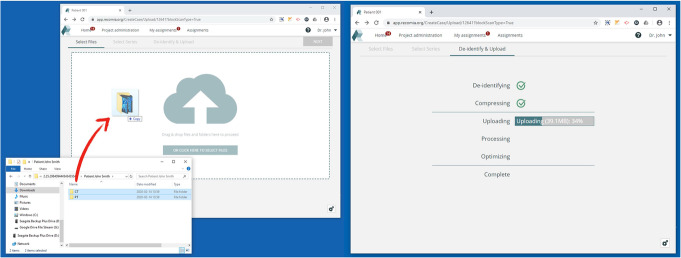
Dicom files using drag-and-drop (left). Before leaving your device, the image files are automatically de-identified (right)

New medical images in DICOM format can be uploaded to the platform using drag and drop. Before leaving your device, the image files are automatically de-identified in accordance with the DICOM standard (Fig. 2). Transfer to the server is secured using the Transfer Layer Security protocol with currently recommended cipher suites.

#### Online viewing and manual segmentation

**Fig. 3:**
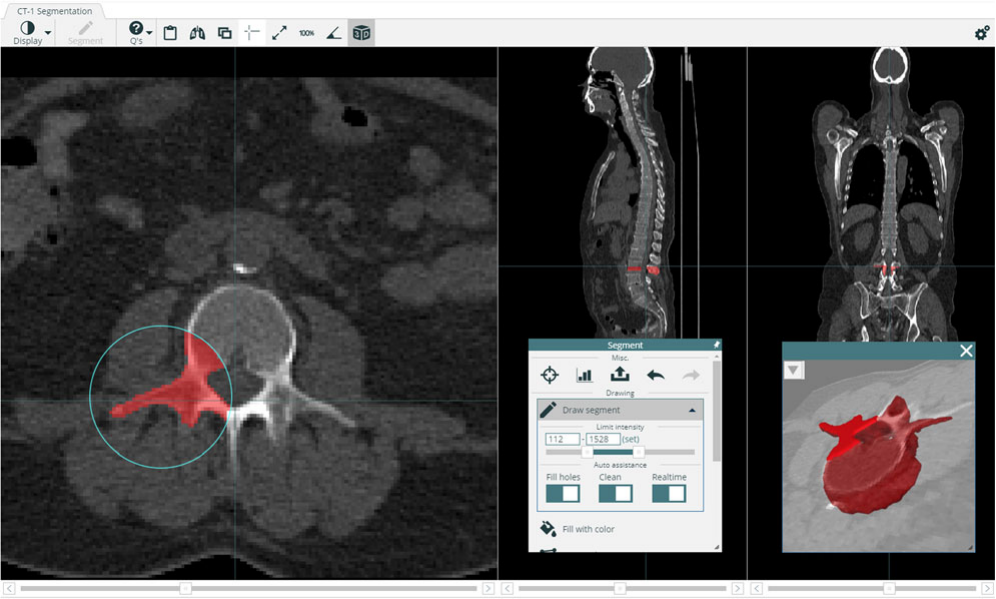
The thresholding brush only paints pixels with Hounsfield values inside a specified range. This can speed up annotation significantly for some organs

The platform also allows viewing and annotating images in the browser. Standard features, such as windowing, zoom, and colour scales for PET studies are available, in similar ways as in conventional workstations. For performing detailed manual segmentations of, for example, organs or lesions, several tools are available. These include basic tools, such as a brush, polygon, and bucket fill tools, but also more advanced tools specialised for medical images (Fig. 3). All tools have full support for multiple labels. Annotation tasks can be administered to different experts via a built-in project management system. Possible tasks include segmenting new labels but also reviewing segmentations performed by other experts. To simplify quality control, it is also possible to view the segmentations in 3D.

The resulting annotations can be saved in separate DICOM files with label information stored in the DICOM file following the DICOM standard.

#### Online AI tools

**Fig. 4:**
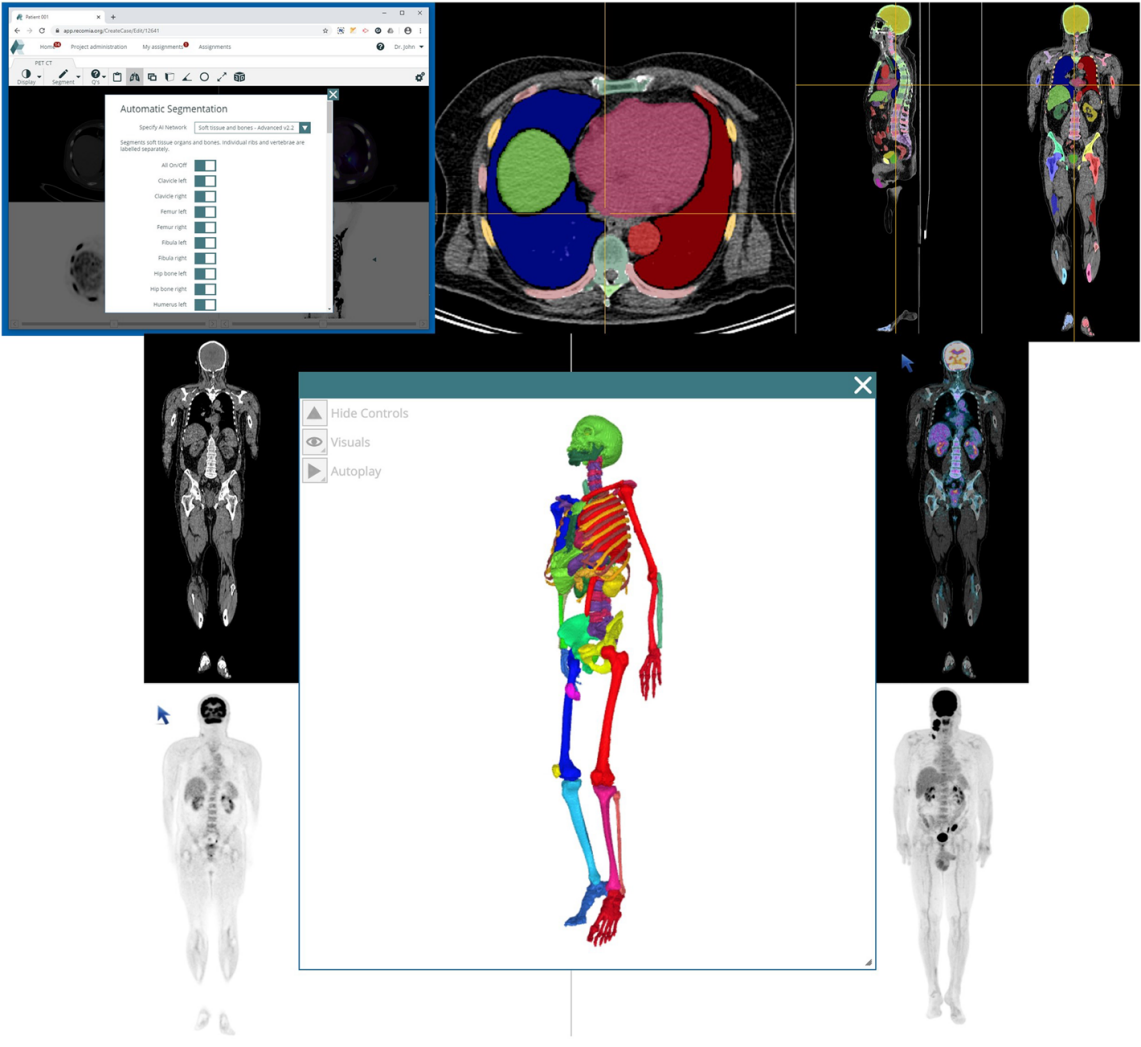
The AI segmentation tool can be used to segment up to 100 different organs (top left). The automated segmentation results can be viewed as an overlay (top right) or in 3D (bottom)

Several deep learning-based tools are already available upon request in the RECOMIA platform, among which the most important is the organ segmentation, described in the next section (Fig. 4). It is also possible to upload your own customised tools and make them available to other researchers. Results from the AI tools can be displayed and corrected if necessary. It is also possible to use the automated results as a starting point for manual annotations.

#### Online quantification

Given a segmentation, whether performed manually or by AI, several statistics are available for each label. This includes the label volume, mean and max pixel values, and the number of connected components. For example, for PET images, this allows the computation of standardised uptake values and total lesion uptakes. The results can be exported as a CSV file.

### Deep learning-based organ segmentation

The RECOMIA platform has allowed the collection of a large dataset of annotated CT and PET/CT images. This data has been used to develop several useful AI tools. Here, we will focus on a tool for organ segmentation. Convolutional neural network (CNN)-based organ segmentation in CT images is already becoming standard, but it is normally limited to segmenting a smaller number of organs [4, 5]. This work takes organ segmentation to the next level by handling 100 different labels, including instance labels, such as vertebrae and ribs, where the number of instances can vary between images.

#### The model

**Fig. 5:**
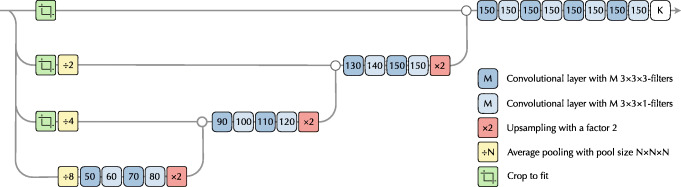
The network structure used for both CNNs. The reason for using two different filter sizes is to compensate for anisotropic voxel sizes and producing an approximately cubic field of view

The organ segmentation tool is based on two CNNs. One CNN handles vertebrae and ribs labels, where there are multiple instances with similar appearances. The other CNN handles all other labels. Both networks are fully convolutional segmentation networks, with structure loosely inspired by the popular U-Net [6], see Fig. 5. Using valid convolutions, the main memory bottleneck during training is the early layers because of their spatial size. By working on four different resolutions, with full field of view only on the lowest resolution, we significantly reduce the memory used during training. In practice, this is implemented using pooling layers at the start of the network. The final convolutional layer contains one channel per label with SoftMax activation.

The instance CNN has three SoftMax output channels coding for background, vertebra, and rib. The receptive field size of the networks is 136 × 136 × 72, approximately corresponding to a cube in millimeters (185 × 185 × 216 mm). This is too small for the instance CNN to predict the correct index of a vertebra. Instead, it has three extra output channels with linear activations. For each foreground pixel, these channels predict the centre of the corresponding vertebra. As a postprocessing step, these coordinates are clustered to identify the individual vertebrae. The final postprocessing step for all labels consists of extracting the largest connected component and filling holes in that component.

Both networks use the same pre-processing; the Hounsfield values are clamped to [− 800, 800] and divided by 800, resulting in an input with values in the range [− 1, 1].

#### Patients and manual segmentations

The CNN-based organ segmentation in CT studies in RECOMIA has been used in multiple studies [7–12]. These studies were approved by the Regional Ethical Review Board (#295/08) and were performed following the Declaration of Helsinki. Patients and image acquisition have been described previously [7, 8, 10, 11].

A group of experienced radiologists and nuclear medicine physicians manually segmented different organs using the RECOMIA platform. The organs included 77 bones and 23 soft tissue organs (Table 1). Not all organs were annotated in all CT studies, which had to be handled in the training process. A dataset of approximately 13,000 manual organ segmentations in 339 images was used to train the CNNs.

**Table 1:** List of the 100 different organs segmented throughout the studies grouped by type

Bones	Organs	Soft tissue	Organs
Skull	1	Adrenal gland	2
Mandible	1	Brain	1
Cervical vertebrae	7	Lungs	2
Thoracic vertebrae	12	Trachea	1
Lumbar vertebrae	5	Bronchi	2
Ribs	24	Heart	1
Sacrum and coccyx	1	Aorta	1
Hip bones	2	Ventricle	1
Scapulae	2	Gastrointestinal tract	1
Clavicles	2	Liver	1
Sternum manubrium	1	Gallbladder	1
Sternum body	1	Spleen	1
Humerus	2	Pancreas	1
Radius	2	Kidneys	2
Ulna	2	Urinary bladder	1
Hand	2	Prostate	1
Femur	2	Testes	1
Tibia	2	Musc. gluteus maximus	2
Fibula	2		
Patella	2		
Foot	2		
**Total**	**77**		**23**

A separate test set of 10 patients (5 male/5 female) was used to test the method and obtain data on inter-observer variability. Each test case was segmented independently by two different readers. Ten organs (prostate only for male patients) were segmented in each CT study.

All images used for training, validation, and test had a pixel spacing of 1.36 mm in slices and a distance between slices of 3 mm. Images with different pixel spacing can still be segmented by resampling the images using trilinear interpolation before running the networks. The resulting segmentation is then resampled to the image resolution using the nearest neighbour interpolation.

#### Training the networks

The annotated data was divided with 80% in a training set and 20% in a validation set used to control hyperparameters. In theory, training a CNN is a simple question of feeding examples to the backpropagation algorithm. In this case, this means feeding randomly selected patches from images in the training group. These patches were augmented using moderate rotations (− 0.15 to 0.15 radians), scaling (− 10 to + 10%), and intensity shifts (− 100 to +100 HU) to enrich the training data. The model was trained using patches of size 136 × 136 × 72 and a batch size of 50. Categorical cross-entropy was used as the loss function, and the optimisation was performed using the Adam method [13] with Nesterov momentum. The networks were developed in Python using the Tensorflow and Keras frameworks. Training and execution were performed on a high-end Linux desktop computer with a GeForce RTX 2080 TI graphics card. The training time for each network was about 48 h. Running the model on a single image took about 2 min on average.

### Statistical methods

The CNN-based segmentation was compared to the manual segmentations. The Sørensen-Dice (Dice) index was used to evaluate the agreement between automated and manual segmentations by analysis of the number of overlapping voxels.

## Results

The model was compared to the manual segmentations (e.g. Fig. 4) on the validation set and the test set. For all foreground pixels in the validations set, the Dice index was 0.95, recall 0.96, and precision 0.94. Another way to measure accuracy is by the per-organ Dice index. The average Dice index over the 100 organs was 0.88 (0.84 for the soft tissue organs and 0.90 for bones). Per organ metrics are shown in Table 2. For the test set, one of the manual segmentations was randomly chosen to be ground truth. The automatic segmentation had foreground Dice index of 0.93, recall of 0.93, and precision of 0.92. The average Dice index over the 10 organs was 0.86 (0.82 for soft tissue organs and 0.90 for bones). Per organ metrics for all organs are shown in Table 3. The inter-observer foreground Dice index, recall, and precision were all 0.94. The average Dice index over the 10 organs was 0.89 (0.86 for soft tissue organs and 0.92 for bone). Per organ metrics for all organs are shown in Table 4.

**Table 2:** Dice index, recall, and precision per organ. Each organ is individually segmented (i.e. the model segmented 24 individual ribs). The metric is presented as the mean over all organs in the same group. The metric for each organ in each group is very similar

Organ	Dice	Recall	Precision
Skull	0.93	0.94	0.92
Mandible	0.90	0.97	0.85
Cervical vertebrae	0.88	0.88	0.88
Thoracic vertebrae	0.91	0.91	0.90
Lumbar vertebrae	0.91	0.91	0.91
Ribs	0.88	0.92	0.85
Sacrum and coccyx	0.94	0.96	0.92
Hip bones	0.96	0.97	0.94
Scapulae	0.95	0.97	0.93
Clavicles	0.94	0.98	0.90
Sternum manubrium	0.93	0.96	0.90
Sternum body	0.92	0.96	0.89
Humerus	0.92	0.95	0.89
Radius	0.94	0.96	0.92
Ulna	0.93	0.98	0.89
Hand	0.87	0.91	0.84
Femur	0.96	0.96	0.97
Tibia	0.96	0.97	0.96
Fibula	0.96	0.96	0.95
Patella	0.96	0.97	0.95
Foot	0.95	0.95	0.96
Adrenal gland	0.61	0.74	0.58
Brain	0.98	0.99	0.96
Lungs	0.98	0.98	0.98
Trachea	0.89	0.91	0.86
Bronchi	0.77	0.86	0.71
Heart	0.92	0.93	0.92
Aorta	0.87	0.88	0.87
Ventricle	0.85	0.88	0.84
Gastrointestinal tract	0.86	0.85	0.89
Liver	0.96	0.97	0.96
Gallbladder	0.78	0.86	0.75
Spleen	0.89	0.93	0.88
Pancreas	0.57	0.68	0.53
Kidneys	0.91	0.95	0.89
Urinary bladder	0.83	0.88	0.81
Prostate	0.82	0.84	0.83
Testes	0.58	0.55	0.66
Muscle gluteus maximus	0.93	0.93	0.92
Average	0.88	0.91	0.87

**Table 3:** Mean Dice index, recall, and precision per organ on an independent test set of 10 patients (5 male/5 female)

Organ	Dice	Recall	Precision
Hip bone left	0.94	0.95	0.94
Humerus left	0.88	0.94	0.84
Rib right 5	0.88	0.91	0.84
Scapula right	0.91	0.92	0.90
Lumbar vertebrae 3	0.89	0.88	0.90
Aorta	0.87	0.91	0.84
Kidney left	0.92	0.94	0.91
Liver	0.95	0.94	0.95
Prostate	0.81	0.93	0.72
Trachea	0.89	0.89	0.88
Average	0.90	0.92	0.87

**Table 4:** Inter-observer Dice index, recall, and precision for the two readers, per organ on an independent test set of 10 patients (5 male/5 female)

Organ	Dice	Recall	Precision
Hip bone left	0.96	0.94	0.97
Humerus left	0.92	0.93	0.92
Rib right 5	0.90	0.89	0.91
Scapula right	0.93	0.91	0.95
Lumbar vertebrae 3	0.88	0.87	0.89
Aorta	0.89	0.90	0.89
Kidney left	0.94	0.94	0.96
Liver	0.95	0.96	0.94
Prostate	0.84	0.85	0.84
Trachea	0.94	0.92	0.95
Average	0.91	0.91	0.92

## Discussion

AI-based tools can provide highly accurate and reproducible organ segmentation, similar to those obtained manually by radiologists, but much faster (approximate manual segmentation time was 90 min per patient for the 10 organs in the test set). To the best of our knowledge, RECOMIA is the only platform that is freely available for research and can be used to automatically segment a wide selection of organs in CT images and provide PET measurements for the same organs. We continue to train new CNNs to continuously improve performance.

Studying the results in Tables 2 and 3, the automatic organ segmentation achieves high Dice scores for most labels. Unsurprisingly, organs that might have low contrast to the surrounding tissue, such as the pancreas, are assigned lower scores. Also, small organs, such as the testes or the adrenal glands, tend to be assigned lower Dice scores. To understand why, note that the difficult pixels are typically found on the organ boundaries, while pixels inside the organ are easier to classify. The number of boundary pixels increases quadratically with organ size, while the total number of organ pixels increases cubically.

Considering the statistical dispersion of Dice indices, it is typically low for organs with high Dice scores. This means standard deviations between 0.01 and 0.05, excluding one outlier case where considerable image noise around the first thoracic vertebra led to an off-by-one error in the numbering of all the subsequent vertebrae and ribs (although well delineated). For the organs with lower average Dice index listed above, the dispersion was also higher with standard deviations between 0.08 and 0.26. Finally, due to large natural variability, the gallbladder, urinary bladder, and ventricle had high standard deviations (0.08 to 0.17) although the average Dice indices were good.

The RECOMIA platform and the deep learning-based tools for organ segmentations have already been used in several studies. Lindgren Belal et al. [7, 8] used bone segmentation for quantification of bone metastases PET/CT in patients with prostate cancer. The automatically measured tumour burden to bone was associated with overall survival. The intra-observer volume difference for the segmentation of five selected bones was less with CNN-based than a manual approach, for example, Th7 2% volume difference for CNN-based segmentation vs 14% for segmentation performed by a radiologist.

Mortensen et al. [9] and Polymeri et al. [10] used automated segmentation of the prostate. A CNN was trained for automated measurements in [18F]-choline PET/CT scans obtained before radical prostatectomy in patients with newly diagnosed prostate cancer [9]. Automated standardised uptake values from the PET images were obtained for the prostate. Corresponding manual measurements were performed, and the CNN-based and manual measurements were compared with the weighted surgically removed tissue specimens. The automated CNN segmentation and the PET measurements provided similar measurements to manually derived measurements. Polymeri et al. [10] then used the method to explore the potential of automatic PET/CT measurements as prognostic biomarkers. These authors found that automated PET/CT measurements reflecting total lesion uptake were significantly associated with overall survival, whereas age, prostate-specific antigen, and Gleason score were not.

Sadik et al. [11] developed automated segmentation of the liver and thoracic aorta as a first step towards an automated method for evaluating treatment response in patients with lymphoma, since those organs are reference organs in the Deauville 5-point scale. The CNN-method showed good agreement with results obtained by experienced radiologists who had manually segmented the CT images. Ly et al. [12] then used the method to calculate Deauville scores in patients with lymphoma, to compare Deauville scores obtained from different reconstruction methods.

The platform is currently used by research groups from 20 hospitals/universities in 10 countries and includes both CT, PET/CT, and magnetic resonance imaging applications.

The organ segmentations are based on low dose CT without contrast on adult patients. The scope will be expanded to include also CT of diagnostic quality and with contrast. Future work will also include organ segmentation of CT studies from children.

## Conclusion

The paper presents a platform that provides deep learning-based tools that can perform basic organ segmentations in CT, which can then be used to automatically obtain the different measurements in the corresponding PET image. The tools developed in this project are available on request at www.recomia.org for research purposes.

## Data Availability

The www.recomia.org platform is freely available for research.

## Abbreviations

AI: Artificial intelligence
CNN: Convolutional neural network
CT: Computed tomography
Dice: Sorensen-Dice
DICOM: Digital imaging and communications in medicine
PET: Positron emission tomography
RECOMIA: Research Consortium for Medical Image Analysis
